# LPCAT1 promotes brain metastasis of lung adenocarcinoma by up-regulating PI3K/AKT/MYC pathway

**DOI:** 10.1186/s13046-019-1092-4

**Published:** 2019-02-21

**Authors:** Chunhua Wei, Xiaomin Dong, Hui Lu, Fan Tong, Lingjuan Chen, Ruiguang Zhang, Jihua Dong, Yu Hu, Gang Wu, Xiaorong Dong

**Affiliations:** 1 Cancer Center, Union Hospital, Tongji Medical College, Huazhong University of Science and Technology, Wuhan, 430022 China; 2 Murdoch Children’s Research Institute, Melbourne, VIC Australia; 3 Department of Paediatrics, University of Melbourne, Melbourne, VIC Australia; 4 Medical Research Center, Union Hospital, Tongji Medical College, Huazhong University of Science and Technology, Wuhan, 430022 China; 5 Institute of Hematology, Union Hospital, Tongji Medical College, Huazhong University of Science and Technology, Wuhan, 430022 China

**Keywords:** LPCAT1, NSCLC, Brain metastasis, PI3K/AKT pathway, MYC, RNA-sequencing

## Abstract

**Background:**

Brain metastasis (BM) is associated with poor prognosis, recurrence, and death in patients with non-small cell lung cancer (NSCLC). Lysophosphatidylcholine acyltransferase 1 (LPCAT1) has been reported to be involved in the progression, metastasis and recurrence of malignancies. However, the potential role of LPCAT1 in NSCLC remains poorly understood. This study was aimed to identify genes involved in lung adenocarcinoma (LUAD) brain metastasis, and look into the role of LPCAT1 in LUAD progression.

**Methods:**

We used integrative genomic analysis to identify genes involved in lung adenocarcinomas. LPCAT1 expression was evaluated in tumor tissues from LUAD patients and LUAD cell lines. The role of LPCAT1 was subsequently investigated both in vitro and in vivo. The mechanism underlying the involvement of LPCAT1 in LUAD progression was explored with the activator of PI3K/AKT pathway. RNA sequencing was performed to confirm the involvement of LPCAT1 and associated pathway in LUAD brain metastasis.

**Results:**

LPCAT1 was up-regulated in LUAD tissues and cell lines. shRNA-mediated depletion of LPCAT1 not only abrogated cell proliferation, migration and invasion in vitro, but also arrested tumor growth and brain metastases in vivo. Notably, LPCAT1 at least partially influenced LUAD progression through PI3K/AKT signal pathway by targeting MYC transcription. Moreover, expression of LPCAT1 was higher in tissues of LUAD patients with BM than those without BM as revealed by IHC staining, RNA-Sequencing and qPCR analysis. Finally, elevated LPCAT1 expression in patients with lung adenocarcinomas was associated with a poor clinical outcome.

**Conclusions:**

This study showed that LPCAT1 works as a regulator of cell metastasis and may serve as a novel therapeutic target for BM in lung adenocarcinoma.

**Electronic supplementary material:**

The online version of this article (10.1186/s13046-019-1092-4) contains supplementary material, which is available to authorized users.

## Background

Lung cancer represents the leading cause of cancer-related deaths, and non-small cell lung cancer (NSCLC) accounted for about 80% of all lung cancer cases. Brain metastasis (BM) is the main cause of poor prognosis, recurrence and death in NSCLC patients, and approximately 20–40% of these patients eventually developed BM [1]. The post-metastasis survival time of patents with BM is no more than one to two months if untreated [2]. Currently, the treatments for brain metastasis from lung cancer include surgery, radiotherapy, chemotherapy and immunotherapy. Nonetheless, the efficacy of all the treatments remains unsatisfactory. Therefore, further study of biological and molecular mechanism underlying BM in NSCLC and identifying new treatment targets have become an urgent task.

Some previous studies suggested female gender, non-smoking, EGFR mutation and age under 60 years are high-risk factors for brain metastasis of NSCLC with stage IIIB/IV [3, 4]. The mechanism underlying brain metastasis is complicated, implicating tumor cells, revascularization and glial cells and so on. Some investigations demonstrated that the expression of proteins involved in PI3K/AKT signaling pathway was up-regulated in patients with brain metastasis of melanoma, suggesting that PI3K/AKT signaling pathway might be used as a target for the treatment of melanoma and other highly invasive tumors [5]. Currently, multiple studies showed that the PI3K/AKT was involved in brain metastases of breast cancer [6, 7] and melanoma [5, 6]. Several activation-specific protein markers in the PI3K/AKT pathway are elevated in brain metastases other than in extracranial metastases [5]. Inhibition of PI3K/AKT pathway might block brain metastasis of melanoma [6]. Furthermore, MYC is a known master transcription factor that regulates genes essential for cell proliferation, survival and metastasis [8–10]. However, the function of PI3K/AKT pathway and MYC in brain metastases of NSCLC remains poorly understood.

Big data analysis can help us acquire more information about the mechanisms of the development and progression of tumors. By searching tumor-related online databases and examining the gene expression in primary loci and adjacent tissues in healthy subjects and lung-cancer patients, we found that LPCAT1 was highly expressed in pulmonary tissues and its over-expression was correlated with the poor prognosis of NSCLC. LPCAT1 is a cytosolic enzyme that catalyzes the conversion of lysophosphatidylcholine (LPC) into phosphatidylcholine (PC) in remodeling the pathway of PC biosynthesis [11]. To date, LPCAT1 overexpression has been reported in clear cell renal cell carcinoma [11], oral squamous cell carcinoma [12], gastric cancer [13] and breast cancer [14]. LPCAT1 has been found to be a contributor to the progression, metastasis, and recurrence of cancer [11, 12, 14]. However, reports on the role and the underlying mechanism of LPCAT1 in NSCLC have been scanty.

In this study, by searching online databases, we acquired the data concerning the LPCAT1 expression in the tumor tissues from lung cancer patients and in NSCLC cell lines. Moreover, we analyzed LPCAT1 expression in NSCLC cell lines and lung tissues of normal subjects, lung tumor tissues from patients with or without brain metastasis. Our results indicated that LPCAT1 was highly expressed in lung tumor tissues, and the LPCAT1 expression was even higher in lung tissues from lung cancer patients with brain metastasis. Moreover, we examined the functions and signaling pathways associated with LPCAT1 in NSCLC both in vivo and in vitro*.* By employing RNA-Sequencing (RNA-Seq), we confirmed that LPCAT1 was more highly expressed in lung cancer tissues in patients with brain metastasis than in their counterparts without BM. Our results suggested that LPCAT1 might be implicated in the carcinogenesis and brain metastasis of NSCLC. Notably, LPCAT1 might promote the proliferation, migration and invasion of NSCLC cells partially by activating PI3K/AKT/MYC signaling pathway.

## Methods

### Datasets and database used in this study

The Cancer Genome Atlas (TCGA) data regarding lung adenocarcinoma (LUAD) patients, including genomic alterations, gene expression and clinical information were obtained from the TCGA Data Portal website (https://portal.gdc.cancer.gov/projects/TCGA-LUAD) of 140 stage IB LUAD patients and 59 adjacent normal samples. Microarray datasets of LUAD patients for gene expression analysis were acquired from online data repositories (Gene Express Omnibus, GEO) dataset (GSE32863 and GSE7670). Public microarray datasets were retrieved from TCGA and Oncomine database, respectively. The three datasets were used for identification of genes overexpressed in LUAD tissues as compared with adjacent normal tissues. Differentially expressed genes (DEGs) between LUAD tissues and adjacent normal tissues were identified by using Limma package. According to the result of Limma package analysis, genes were filtered and selected if a *P* value was less than 0.01. Funrich Software (Version 3.0, http://funrich.org/index.html) was utilized to analyze the features of DEGs.

The Human Protein Atlas (THPA) (https://www.proteinatlas.org/) is an online database, which includes the human tissues, the human cell, human pathology and protein classes [15, 16]. We used the IHC data from THPA efforts to examine the expression of LPCAT1 in 17 types of major human cancer and the positive rate of LPCAT1 in lung cancer tissues.

Oncomine (https://www.oncomine.org/resource/login.html) is an important data-mining platform [17]. The data concerning clinical stages of lung adenocarcinoma patients were taken from this database.

### Pathway and gene set analysis

The functional analysis was performed by using the Database for Annotation, Visualization and Integrated Discovery (v6.8, DAVID, https://david.ncifcrf.gov/home.jsp) [18, 19].

Gene Set Enrichment Analysis (GSEA) is a method of calculation. To characterize signaling pathways associated with LPCAT1 expression, we performed GSEA by using data from the TCGA cohort of LUAD (*n* = 522). Comparison between mRNA expression and amplification status of LPCAT1 in 522 patients with LUAD showed that 512 cases were successfully matched. LUAD (*n* = 512) patients were divided into 2 groups, with the amplification status of LPCAT1 used as phenotypic labels (LPCAT1 amplification group and LPCAT1 non-amplification group). A ranked list of protein coding gene was obtained and subjected to GSEA analysis for C2 curated gene sets, C4 computational gene sets, C6 oncogenic signatures and KEGG pathways curated gene sets from Broad Institute Molecular Signature Database (MSigDB). As recommended by the GSEA, gene sets with FDR less than 0.25 were considered significant [20].

### Cell lines and reagents

A549, HCC827 cell lines and the normal human bronchial epithelial cell line Beas-2B were obtained from the Institute of Biochemistry and Cell Biology of the Chinese Academy of Sciences, Shanghai, China. H460 and H1975 cell lines were from the American Type Culture Collection (ATCC). PC-9 cells were procured from Cobioer corporation (Nanjing, China). All cells were cultured in RPMI 1640 medium (Gibico, Grand Island, NY, USA) supplemented with 10% fetal bovine serum (10% FBS), 100 U/ml penicillin and 100 mg/ml streptomycin (Invitrogen, Carlsbad, CA, USA) in humidified air at 37 °C with 5% CO_2_.

### Establishment of stable lung cancer cell lines

lentiviral LPCAT1 shRNA and the negative control constructs, which carry the EF1 promoter-driven firefly luciferase and puromycin resistance gene, and the corresponding virus were purchased from GenePharma (Shanghai, China). The titer of lentivirus was determined with the serial dilution method. Then, 1 × 10^8^ TU/ml lentivirus and 2 μg/ml polybrene were used to transduce HCC827 or PC-9 cells seeded in 96-well plates. Cells were incubated in 5% CO_2_ at 37 °C for 24 h. The medium was refreshed and cultured for another 48 h. Stable cell lines were selected by using puromycin. The stably transduced cells were used for all the in vitro and in vivo experiments.

### Cell counting Kit-8 assay

Cells were seeded in 96-well plates (5 × 10^3^cells/well). Cell proliferation was evaluated by cell counting kit-8 (CCK-8, Dojindo Laboratories, Kumamoto, Japan) assay according to the manufacturer’s protocols. Briefly, 10 μl of CCK-8 solution was added to the culture medium and incubated for 2 h in 5% CO_2_ at 37 °C. Then, the absorbance at 450 nm was measured. The cell proliferation was determined on days 1, 2, 3, 4 and 5. All experiments were repeated at least three times.

### Quantitative reverse transcription-polymerase chain reaction (qRT-PCR)

RNA was isolated from blood samples and cells by using TRIzol reagent (Invitrogen, Carlsbad, CA, USA) by following the manufacturer’s instructions. The blood samples were collected and then centrifuged at 1500 rpm for 10 min at room temperature. Serum was transferred into RNA-free EP tubes and stored at − 80 °C before RNA extraction. Complementary DNA (cDNA) was synthesized from total RNA using PrimeScript RT reagent Kit (Takara, Dalian, China), and PCR was performed using SYBR Green RT-PCR Kit (Takara). GAPDH served as an internal control. PCR was run on the StepOne Plus Real-Time PCR System (Applied Biosystems, Foster City, CA, USA), and data were analyzed using the 2^-∆∆CT^ method. Primers used were as follows: LPCAT1, 5’-ACCTATTCCGAGCCATTGACC-3′ (forward), 5’-CCTAATCCAGCTTCTTGCGAAC-3′ (reverse); GAPDH, 5’-AATCCCATCACCATCTTCCAG -3′ (forward), 5’-GAGCCCCAGCCTTCTCCAT-3′ (reverse).

### Western blotting and immunoprecipitation

Cells were lysed using RIPA buffer (Beyotime) supplemented with protease inhibitor cocktail (Thermo Scientific, Waltham, MA, USA) and PMSF. Protein concentration was measured using a BCA method. 30–50 μg cell lysates were subjected to sodium dodecyl sulfate-polyacrylamide gel electrophoresis (SDS-PAGE), transferred to polyvinyldene fluoride (PVDF) membrane (Sigma, St Louis, MO, USA) and incubated with specific primary antibodies. Autoradiograms were densitometrically quantified (Quantity One software; Bio-Rad), with GAPDH serving as internal control. The antibodies used were as follows: LPCAT1 (16112–1-AP, ProteinTech, Chicago, IL, USA), MYC (10828–1-AP, Proteintech), GAPDH (60004–1-Ig, Proteintech), AKT (4691, Cell Signaling Technology, MA, USA), p-AKT (4060, Cell Signaling Technology), PI3K (4249, Cell Signaling Technology), MMP-9 (10375–2-AP, Proteintech).

For immunoprecipitation (IP), to investigate the interaction between LPCAT1 and MYC at the endogenous level, HCC827 cells at 80–90% confluence were washed with ice-cold PBS three times before being lysed in IP lysis buffer. Then the lysates were incubated with anti-LPCAT1 and anti-MYC antibodies separately overnight at 4 °C. Protein A/G-agarose beads were added for 2 h or overnight. The beads were collected and washed with lysis buffer for three times. The precipitated proteins were eluted and denatured in 2 × SDS loading buffer and analyzed by western blotting.

### Flow cytometry of cell cycle

Cells were harvested and fixed in 80% ice-cold ethanol in PBS after washing in ice-cold PBS. Then cells were incubated at 37 °C for 30 min and then bovine pancreatic RNAase (Sigma) was added at a final concentration of 2 mg/ml and 20 mg/ml of propidium iodide (PI, Sigma-Aldrich) for 30 min at room temperature. Cell cycle distribution was flow cytometrically determined using a FACScan (Becton Dickinson, Franklin Lakes, NJ, USA). All experiments were conducted in triplicate.

### Transwell migration and invasion assays

For the migration assay, 5 × 10^5^ cells in 200 μl of serum-free medium were placed into the top chamber of a transwell chamber (8 μm pore size, BD Biosciences, San Jose, CA, USA), for the invasion assays, 1.5 × 10^6^ cells were transferred onto the upper chamber coated with Matrigel (BD Biosciences), lower chamber containing 600 μl of 10% FBS medium, serving as a chemoattractant. After 24 h of incubation, cells remaining inside the upper chambers were removed while cells attached to the lower surface of the membrane were fixed and stained with Crystal violet (Sigma). Afterwards, cells were imaged and counted under an IX71 inverted microscope (Olympus, Tokyo, Japan).

### Wound-healing assay

For the wound-healing assay, cells were seeded into 6-well plates (1 × 10^5^ cells/well). The monolayer was scratched with a 10 μl plastic pipette tip to create a uniform wound. Then, the monolayer was washed with PBS and incubated in culture medium without fetal bovine serum (FBS). The wound margin distance between the two edges of the migrating cell sheets was photographed after scratching under a phase-contrast microscope. All experiments were performed in triplicate.

### In vivo xenograft assay

Tumor cells were prepared by suspending 6 × 10^6^ HCC827 and PC-9 cells in 100 μl of serum-free medium, and inoculated onto right rear flanks of 4-week-old female BALB/c nude mice (Beijing Huafukang Bioscience Company, Beijing, China). The tumor growth was monitored and recorded on weekly basis after the inoculation. Tumor volume was calculated as follows: 0.5 × tumor length × tumor width^2^. In accordance with institutional guidelines on animal care, experimental endpoints were determined by one of the followings: (1) tumor size exceeding 2 cm in any dimension, or (2) development of further complications affecting animal welfare. Upon reaching experimental endpoints, mice were humanely euthanized, and tumors were excised and dissected for characterization and further studies. All animal experiments were performed in accordance with a protocol approved by the Institutional Animal Care and Use Committee of Huazhong University of Science and Technology.

### In vivo model of brain metastasis

In vivo model of brain metastasis was described previously [21]. Tumor cells (3 × 10^5^ in 0.1 mL PBS) were slowly injected into the intracarotid artery of nude mice. Bioluminescence imaging was conducted on the 50th days, after tumor cell inoculation or when they seemed to be moribund or when clinical symptoms of brain metastases, such as immobility, weight loss, or a hunched position, developed. Each mouse was imaged using IVIS Lumina imaging system (Calipers, Hopkinton, MA, USA) after intraperitoneal injection of 150 mg/kg luciferin (Goldbio., St. Loius, MO, USA) for 10 min. Their brains were then removed and cut into 2 - to 3 - mm sections. The presence of brain metastases was histopathologically confirmed.

### Immunohistochemical analysis

After dewaxing in xylene and rehydration in graded alcohols, tissue sections were boiled in citrate buffer, pre-incubated with H_2_O_2_, and blocked with rabbit or goat serum (DAKO, Glostrup, Denmark). Sections were then incubated with a primary antibody and then with an HRP-conjugated secondary antibody. Proteins of interest were visualized using diaminobenzidine before counterstaining with hematoxylin. Antibodies used were as following: LPCAT1 (16112–1-AP, Proteintech), PCNA (10205–2-AP, Proteintech), CD34 (ab81289, Abcam, Cambridge, MA, USA) and MMP9 (10375–2-AP, Proteintech).

### Patients selected for RNA-sequencing study

A total of 6 patients with histopathologically confirmed NSCLC (against AJCC criteria) were enrolled in the RNA-Seq study, 3 patients with BM and other 3 without BM. This study was approved by the Institutional Review Board of Huazhong University of Science and Technology, Wuhan, China. Written informed consents were obtained from all patients. BM was established by certified oncologists on the basis of whole brain MRI.

### RNA isolation and cDNA library preparation for RNA sequencing

All the cancer tissues used in this study were taken from surgical specimens and biopsies. Tissue specimens were dissected and preserved immediately in liquid nitrogen after surgery. RNA isolation, cDNA library construction and RNA sequencing were performed by Wuhan Kingstar Medical Inspection Company, Wuhan, China. Briefly, total RNA was extracted from tissue by using TRIzol reagent (Invitrogen) and the quality of extracted RNA was assessed by using Bioanalyzer (Agilent, Waldbronn, Germany). Total RNA samples were treated with DNase I to remove potential genomic DNA and the polyadenylated fraction of RNA was isolated for RNA-Seq library preparation. Truseq Stranded mRNA Sample Prep Kit (Illumina, San Diego, California, USA) was used to construct the stranded libraries by following the manufacturer’s instructions. All libraries were sequenced on the Illumina Hiseq 2500 Sequencer (Novogene Bioinformatics Technology Co., Ltd., Beijing, China).

### RNA sequencing data analysis

For RNA-Seq analysis, the 100 bp stranded paired-end reads were mapped to the human reference (GRCh38) using TopHat (version 2.0.11) and Bowtie2 (version 2.2.2). Cufflink was used to assemble mapped reads based on reference annotation file and FPKM (Fragments per kilobase of transcript per million mapped reads) value were calculated for annotated genes. HTSeq-count was used to count reads of annotated genes for differential expression analysis. Differentially expressed genes between lung tumor tissues with brain metastasis group (BM+) and lung tumor tissue without brain metastasis group (BM-) were identified by DESeq2 R package. According to the result of DESeq2 analysis, genes were classified as differentially expressed when fold changes were more than two and *P value* of statistical test less than 0.05. Funrich Software (Version 3.0, http://funrich.org/index.html) was utilized to analyze the overlaped DEGs among the three online datasets and RNA-Seq results in this study. The RNA-Seq datasets are being deposited in Gene Expression Omnibus (GEO) under the accession number GSE126548.

### Statistical analysis

Data from GEO and TCGA database were processed as aforementioned. Results were reported as the mean ± SD. Comparisons between two groups were made by using the unpaired *t* test. Differences among multiple groups were determined by one-way ANOVA with *post-hoc* Tukey HSD test. A *P* < 0.05 was considered to be statistically significant.

## Results

### Integrative analysis for the identification of genes involved in LUAD

**Fig. 1:**
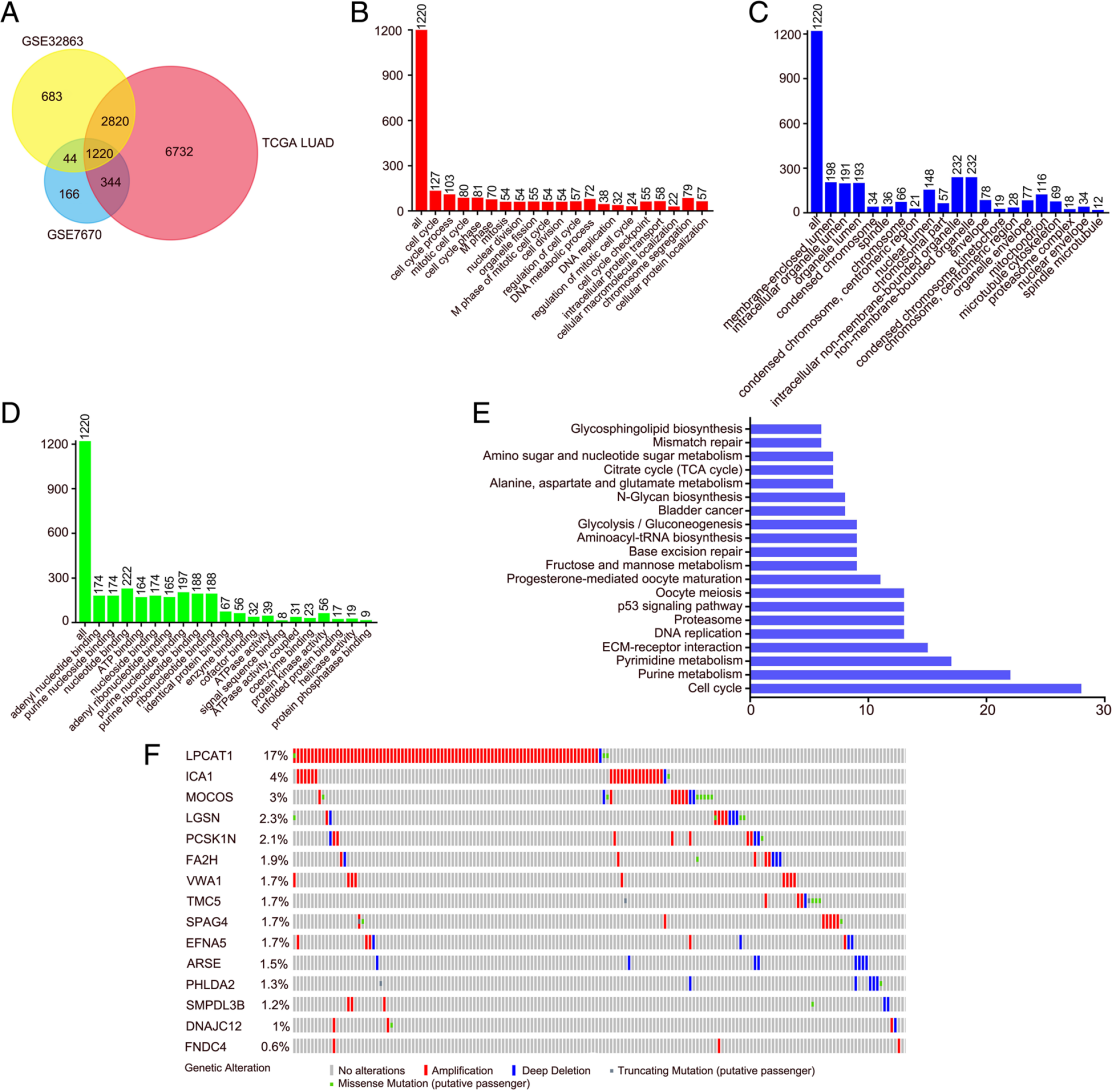
LPCAT1 was identified as an upregulated gene in LUAD tissues by integrative analysis. **a** Venn chart identified 1220 upregulated genes in LUAD tissues in comparison to normal tissues from three online datasets (GSE32863, GSE7670 and LUAD dataset from the TCGA). **b**, **c**, **d** All these 1220 genes were subject to GO term analysis. **e** Enriched KEGG pathway analysis of 1220 genes by DAVID. **f** Genetic alterations of 15 candidate genes were infrequent in 522 LUAD patients. The TCGA database was analyzed for DNA amplifications, deep deletions, truncating mutations and missense mutations

Given the lack of early detection and effective therapies of LUAD at the early stages, it is important to understand its underlying mechanism. Hence, our efforts were made for comprehensive, cross-platform integrated analysis of LUAD, with the aim of unveiling the mechanisms involved in LUAD and identifying new therapeutic targets. We tried to find candidate genes involved in lung cancer by analyzing GEO dataset (GSE32863 and GSE7670) and LUAD dataset from the TCGA. The differentially expressed genes from LUAD patients and normal patients in these three datasets were identified by using limma package, respectively (Additional file 1: Table S1). As shown in Venn diagram, 1220 differentially-expressed genes (Additional file 2: Table S2) were significantly up-regulated in lung adenocarcinoma tissues among three datasets in comparison to normal tissues (Fig. 1a). Then, we analyzed the functions of these 1220 up-regulated genes by using DAVID. The top 20 GO terms related to biological processes, molecular functions and biological cellular components are shown in Fig. 1b-d and Additional file 3: Table S3. The predominantly enriched biological process included ‘regulation of cell cycle’ (GO: 0051726) and ‘cell division’ (GO: 0051301). In terms of cellular components, ‘organelle lumen’ (GO: 0043233) and ‘condensed chromosome’ (GO: 0000793) were the most-enriched subcategories. These genes were also enriched in molecular functions included ‘ATP binding’ (GO: 0005524) and ‘nucleotide binding’ (GO: 0000166). KEGG pathway analysis showed that signaling pathways involved in cell cycle, bladder cancer and p53 signaling pathway were enriched. All these results indicated that these genes may be related to the occurrence of cancer (Fig. 1e).

So far, we found the 15 candidate genes in LUAD patients were largely unexplored among identified genes involved in LUAD. PHLDA2 (Pleckstrin Homology Like Domain Family A Member 2) is believed to be an important oncogenic gene in LUAD [22]. Both ephrins (EFNs) and their receptors (Ephs) are membrane-bound, restricting their interaction with the sites of direct cell-to-cell interfaces. All EFNAs induce epidermal differentiation markers and suppress cell adhesion genes [23]. EFNA5 (Ephrin A5) showed significantly higher expression in prostate tumors than in normal prostate tissue, and might plays an important role in the development of prostate cancer [24]. To find if gene expression is associated with copy number alteration, we further examined the genomic alternations in these 15 candidate genes by analyzing genomic dataset of 522 LUAD patients from TCGA. DNA copy number variant analysis identified genomic gains (amplification) and losses (deletion). We found LPCAT1 amplification in 17% of the patients, which was the most frequent event among the 15 candidate genes (Fig. 1f). These observations indicated that LPCAT1 might be highly expressed in LUAD patients.

### LPCAT1 was essential for the proliferation, migration and invasion of NSCLC in vitro

**Fig. 2:**
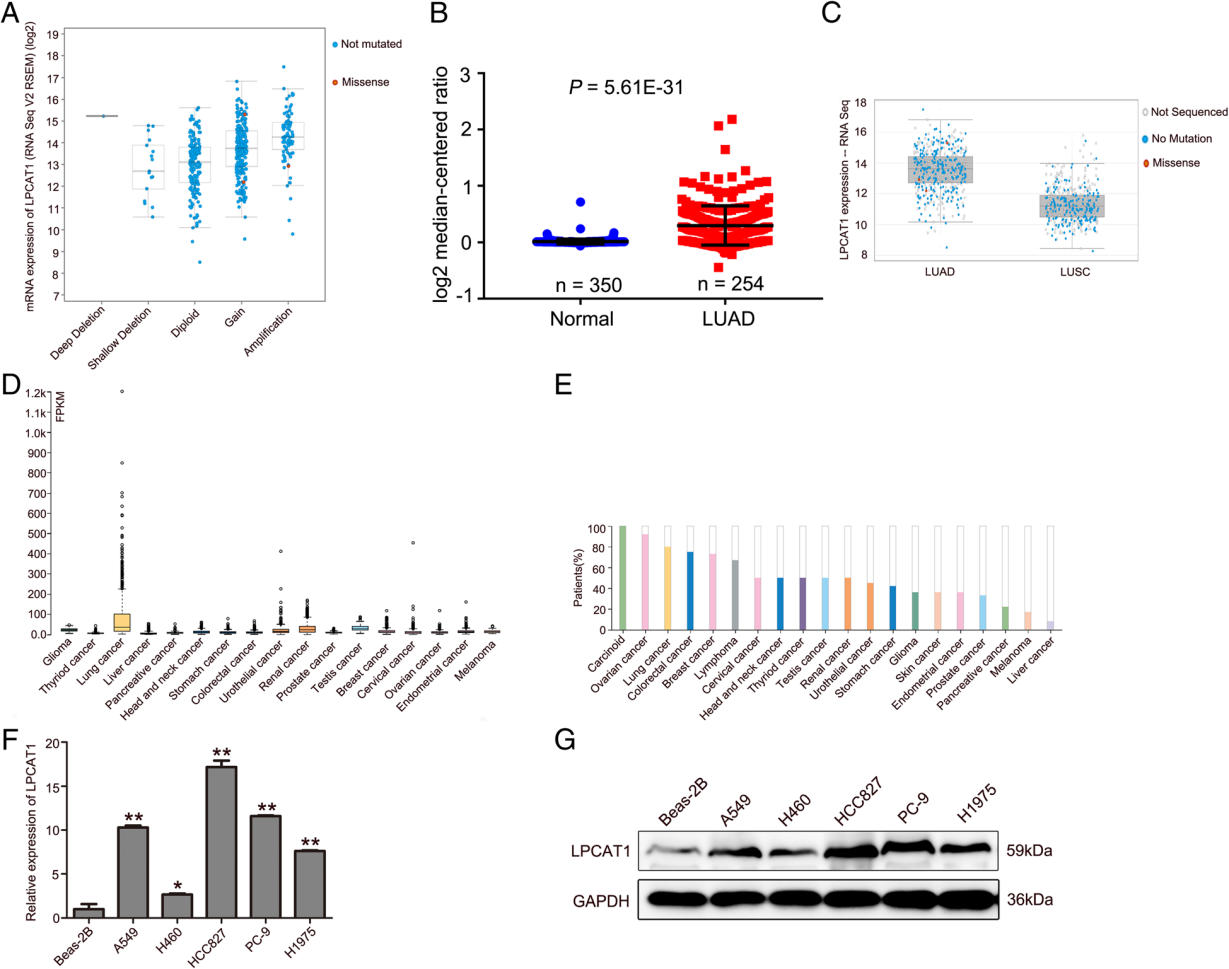
LPCAT1 was upregulated in various cancer patients, especially in lung cancer. **a** The copy number of LPCAT1 was directly proportional to the amount of mRNA expressed in the TCGA database. Blue and red circles represent non-mutated and missense genes, respectively. **b** LPCAT1 expression levels in normal lung tissues (left, *n* = 350) and LUAD tissues (right, *n* = 254) based on TCGA data. Statistical analysis (One-way ANOVA). **c** The expression of LPCAT1 in LUAD (left) was significantly higher than in LUSC (right) based on TCGA data. Black, blue and red circles represent not sequenced, no mutation and missense genes, respectively. **d** Analysis of the THPA database showed that the LPCAT1 expression was relatively higher in patients with primary lung cancer than in those with other 16 tumors. **e** The positive rate of LPCAT1 was up to 80% in lung cancer tissues based on the THPA database. **f**, **g** The expression of LPCAT1 in NSCLC cell lines was detected by PCR and Western blotting. Data are expressed as mean ± SD, *P < 0.05, **P < 0.01, one-way ANOVA

Given that substantially higher LPCAT1 expression in LUAD tissues than in normal lung tissues according to TCGA LUAD and GEO datasets (GSE32863 and GSE7670), we first looked into whether the elevated expression of LPCAT1 is associated with the development of NSCLC. Analysis of the TCGA datasets revealed that the copy number of LPCAT1 was directly proportional to its mRNA expression (Fig. 2a). Moreover, the expression of LPCAT1 in LUAD was significantly higher than in normal lung tissues and in lung squamous cell carcinoma (Fig. 2b and c). Additionally, we searched the THPA database to further examine the expressions of LPCAT1 in patients with various cancers. We found that LPCAT1 expression was relatively higher in patients with lung cancers than in those with other 16 tumors (Fig. 2d). Moreover, search of the THPA dataset showed the positive rate of LPCAT1 was up to 80% in lung cancer tissues (Fig. 2e). Together, these findings suggested LPCAT1 level increased in NSCLC tissues. Next, we performed PCR and Western blotting to assess LPCAT1 expression in NSCLC cell lines. As expected, both LPCAT1 mRNA expression and protein expression were found to be highly expressed in NSCLC cell lines (Fig. 2f and g).

**Fig. 3:**
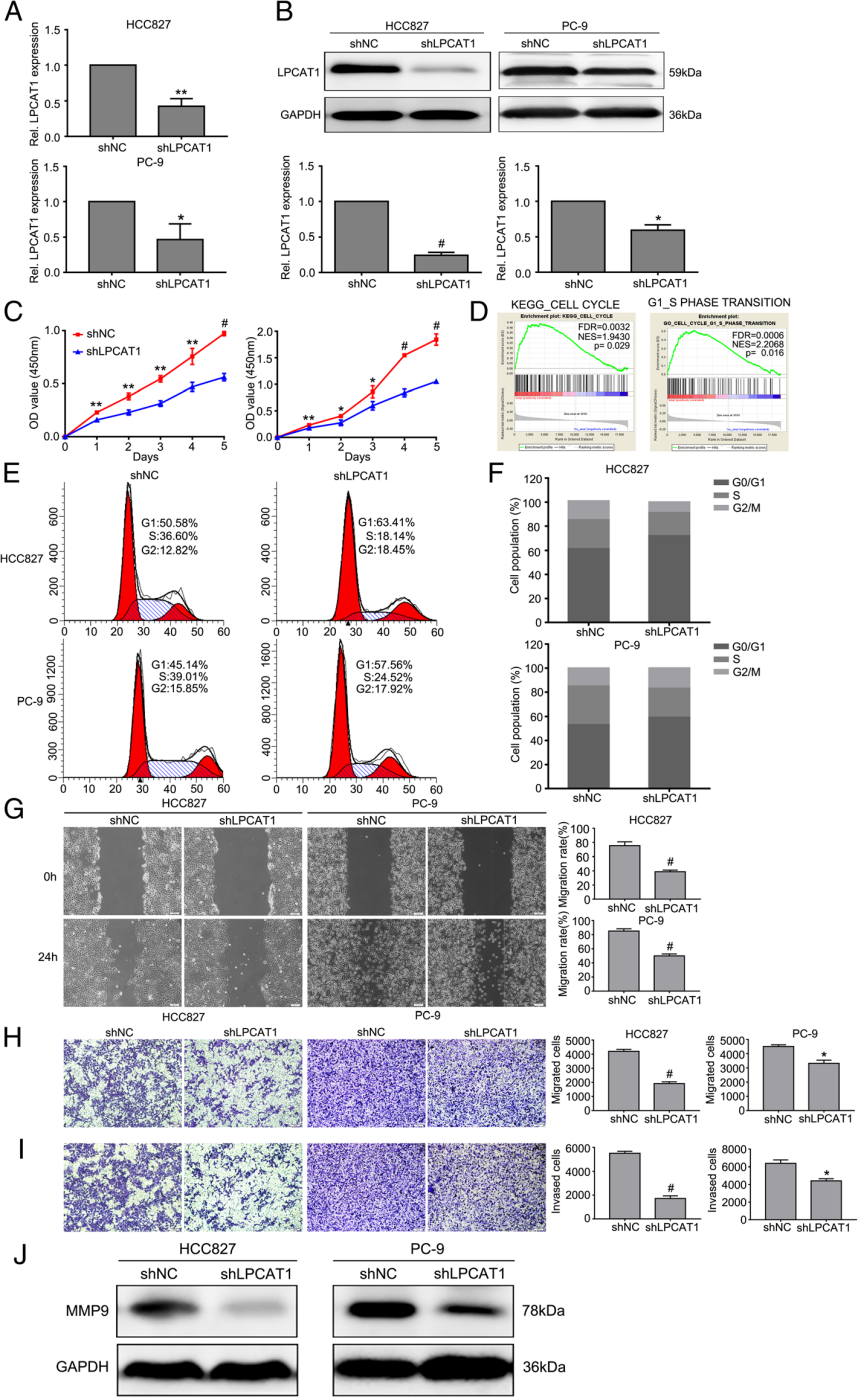
Knockdown of LPCAT1 inhibited the proliferation, migration and invasion of NSCLC cells. **a, b** PCR and Western blotting showed the expression of LPCAT1 in HCC827 and PC-9 cells stably transfected with shNC and shLPCAT1. **c** The proliferative ability of HCC827 and PC-9 cells after transfection was evaluated by CCK-8 assay. **d** GSEA analysis showed that the LPCAT1 amplification status was positively correlated with genes related to “KEGG CELL CYCLE” and “GO CELL CYCLE G1-S TRANSITION”. **e**, **f** The cell cycle was flow cytometrcally studied after PI staining, and the data were processed with ModFit LT program. Down-regulation of LPCAT1 induced G1 phase arrest. **g** Wound-healing assays were performed to assess NSCLC cells migration. Wound closure was determined 24 h after the scratch. **h**, **i** Representative images and quantification of transwell assays indicated the migration and invasive capability of NSCLC cells stably transfected with shNC and shLPCAT1. **j** The expression of MMP-9 was determined by Western blotting. Data are presented as mean ± SD, *P < 0.05, **P < 0.01, t test

Patients with EGFR mutation were reportedly susceptible to BM [25]. Thus, we used HCC827 cell lines with EGFR mutation at exon 19 and PC-9 cell line with EGFR mutation at exon L858R to investigate the function of LPCAT1. We found that LPCAT1 expression was higher in those two cell lines than in other NSCLC cell lines. HCC827 and PC-9 cell lines were then transduced with lentivirus bearing shRNA to knockdown LPCAT1 expression. Efficient depletion of LPCAT1 expression was confirmed by PCR and Western blotting (*P* < 0.05, Fig. 3a and b). We then studied the effect of LPCAT1 on NSCLC cell proliferation in vitro. As a result, the CCK-8 assay revealed that knockdown of LPCAT1 in both cells significantly inhibited the ability of cell proliferation, as compared to that in the shNC cells (*P* < 0.05, Fig. 3c). Since GSEA analysis showed that LPCAT1 amplification was positively correlated with genes related to “KEGG CELL CYCLE” and “GO CELL CYCLE G1-S TRANSITION” (Fig. 3d), we further examined the effect of LPCAT1 on cell cycle regulation of NSCLC cells. Flow cytometry showed that down-regulated expression of LPCAT1 induced G1 phase arrest, suggesting it is involved in cell cycle regulation (*P* < 0.05, Fig. 3e and f). Collectively, these results indicated that suppression of LPACT1 inhibited NSCLC cell proliferation by arresting NSCLC cell cycle in G1 phase.

Since tumor cell metastasis is an important part of tumor progression, we raised a question whether LPCAT1 is involved in the metastasis of NSCLC cells. To answer the question, we performed the wound healing assay and transwell analysis to examine the effect of LPCAT1 on cell migration and invasion. We found that LPCAT1 knockdown inhibited cell migration and invasion of HCC827 and PC-9 cells (Fig. 3g-i). Since MMP-9 expression was associated with metastatic potential [26], we examined the expression of MMP-9 by Western blotting, and found that loss of LPCAT1 inhibited MMP-9 expression (Fig. 3j). Taken together, these findings suggested that suppression of LPCAT1 inhibited migratory and invasive behaviors of NSCLC cells.

### LPCAT1 promoted tumorigenesis and brain metastasis in vivo

**Fig. 4:**
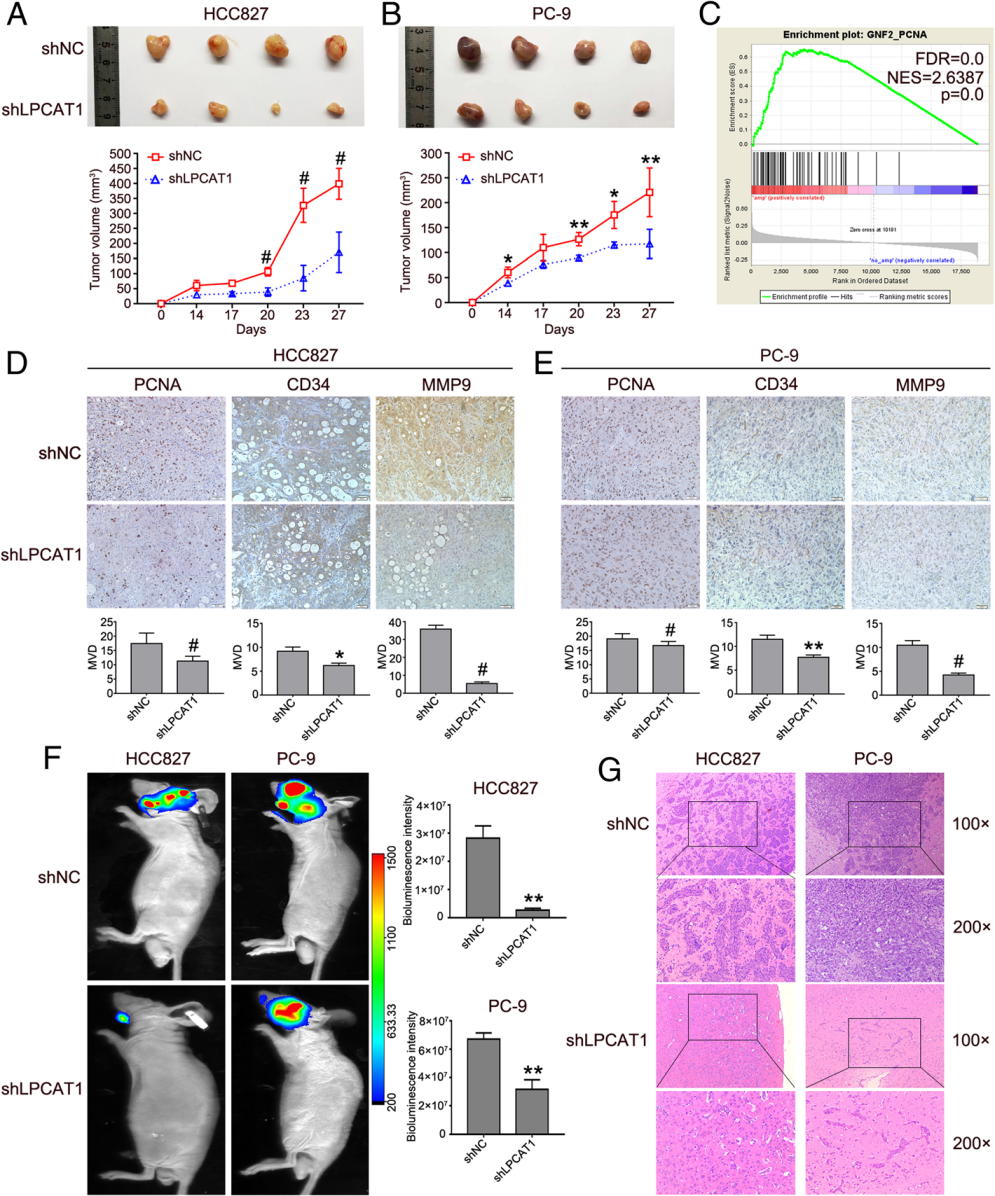
Depletion of LPCAT1 inhibited the tumorigenesis and brain metastasis in vivo. **a**, **b** HCC827 and PC-9 cells stably transfected with NC and shLPCAT1 were injected subcutaneously into nude mice. Four weeks after the injection, mice were photographed and killed. Tumor growth curves were plotted. **c** Gene set enrichment analysis (GSEA) showed that amplification of LPCAT1 was correlated with the geneset of “GNF2_PCNA” in LUAD patients. **d**, **e** Representative IHC staining and quantitative analysis of PCNA, CD34 and MMP9 in xenograft tumors. **f**, **g** Representative bioluminescent/photographic images (**f**) and HE images (100×, 200×, **g**) in brain metastatic mice. The data are expressed as the mean ± SD, *P < 0.05, **P < 0.01, ^#^ *P* < 0.001, as determined by the *t*-test

To further characterize the effect of LPCAT1 knockdown on NSCLC growth in vivo, the xenograft model and brain metastases model were established by implantation of stably-transfected HCC827 and PC-9 cell lines without or with reduced LPCAT1 expression by lentivirus-mediated shRNA knockdown. The result indicated that tumor growth was substantially inhibited in LPCAT1 depletion group, as shown by smaller tumor size and lighter tumor weight (Fig. 4a and b). GSEA analysis of LUAD datasets showed that amplification of LPCAT1, in LUAD patients, was correlated with the gene-set of “GNF2_PCNA” (Fig. 4c). To characterize the features of tumor xenograft model, we performed IHC staining for cell proliferation marker PCNA, microvessel density marker CD34 and metastasis-associated marker MMP-9 in xenograft tissues. Consistent with tumor growth inhibition, the result showed that in tumors with depleted LPCAT1 the expression of PCNA, CD34 and MMP9 were diminished (*P* < 0.05, Fig. 4d and e and Additional file 4: Figure S1A). Since brain was the target organ where NSCLC tends to metastasize, we further explored the effects of LPCAT1 on brain metastasis. Our result indicated that the mice were burdened with more brain metastatic lesions in shNC group than in LPCAT1 depletion group (Fig. 4f and g and Additional file 4: Figure S1B). Together, these results showed that blockade of LPCAT1 activation suppressed tumor growth and brain metastasis of NSCLC cells in vivo.

### LPCAT1 knockdown attenuated the PI3K/AKT signaling pathway at least in part by targeting MYC

**Fig. 5:**
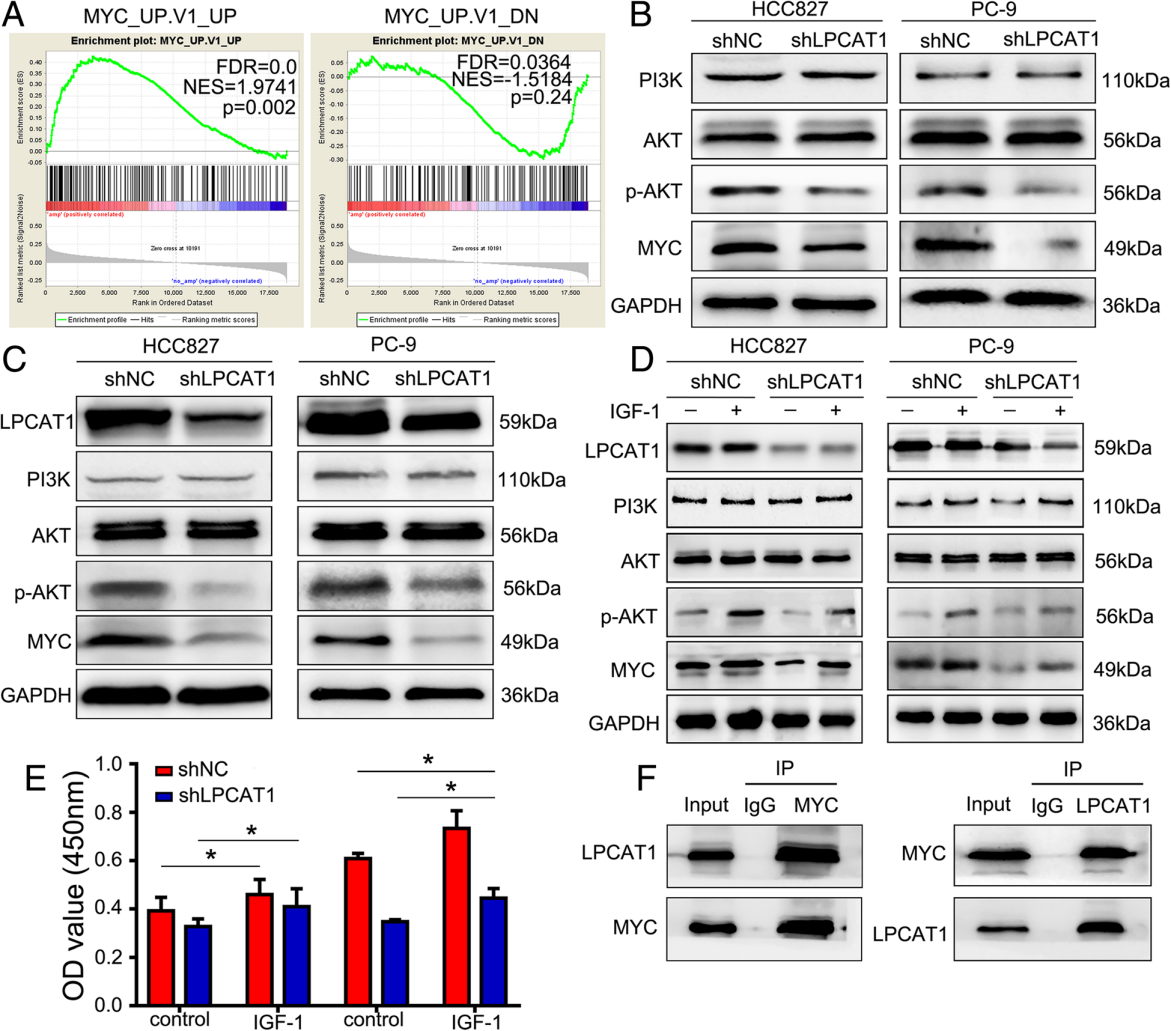
Knockdown of LPCAT1 attenuated the PI3K/AKT signaling pathway at least in part by targeting MYC. **a** GSEA analysis showed that LPCAT1 amplification status was correlated with MYC-activated target geneset and negatively correlated with MYC-suppressed target gene set. **b** The expression of indicated molecules was determined by Western blotting. **c** Mice were sacrificed after 4 weeks and tumor lesions were harvested. The protein lysates extracted from the tumors were used for Western blotting to detect the expression of the indicated molecules. **d** HCC827 and PC-9 cells were incubated with IGF-1 (2 μg/ml) for 24 h and the cell lysates were collected. The expression of indicated molecules was determined by Western blot analysis. **e** After incubation with IGF-1 (2 μg/ml) for 24 h, the cell activity was detected by CCK-8 assay. **f** Co-IP and Western blotting indicated the endogenous interaction between LPCAT1 and MYC protein in HCC827 cells. Data shown were the average of three independent experiments with similar results. The data are presented as the mean ± SD, *P < 0.05 as determined by the *t*-test

To explore the mechanism underlying the involvement of LPCAT1 in tumor metastasis, we set out to identify the relevant signaling pathways accounting for tumor growth inhibition caused by LPCAT1 knockdown. MYC plays an important role in cancer progression [27]. Importantly, GSEA analysis showed LPCAT1 amplification status was correlated with MYC-activated target gene set and negatively correlated with MYC-suppressed target gene set (Fig. 5a). We then assessed the MYC protein levels by Western blotting in the NSCLC cells and xenograft tissues. Notably, loss of LPCAT1 inhibited the expression of p-AKT and MYC in vitro (Fig. 5b) and in vivo (Fig. 5c), suggesting that the PI3K/AKT/MYC pathway was involved in the inhibition of NSCLC metastasis caused by LPCAT1 reduction. Previous studies showed that the cytokine IGF-1 could upregulate the PI3K/AKT pathway [28, 29]. To further confirm these findings, we treated the cells with exogenous IGF-1 (2 μg/ml) for 24 h, and found that the IGF-1 reversed the reduced expression of p-AKT and MYC induced by LPCAT1 knockdown (Fig. 5d). CCK-8 assay showed that the proliferation of LPCAT1 knockdown cells was increased upon treatment with exogenous IGF-1 (Fig. 5e). Co-IP assay indicated that LPCAT1 could interact with MYC (Fig. 5f). These results demonstrated that LPCAT1 promoted the progression of NSCLC cells possibly partially by activating PI3K/AKT/MYC signaling pathway and interacting with MYC.

### Lung tumors from patients with BM had high LPCAT1 expression and PI3K/AKT/MYC gene signatures

**Fig. 6:**
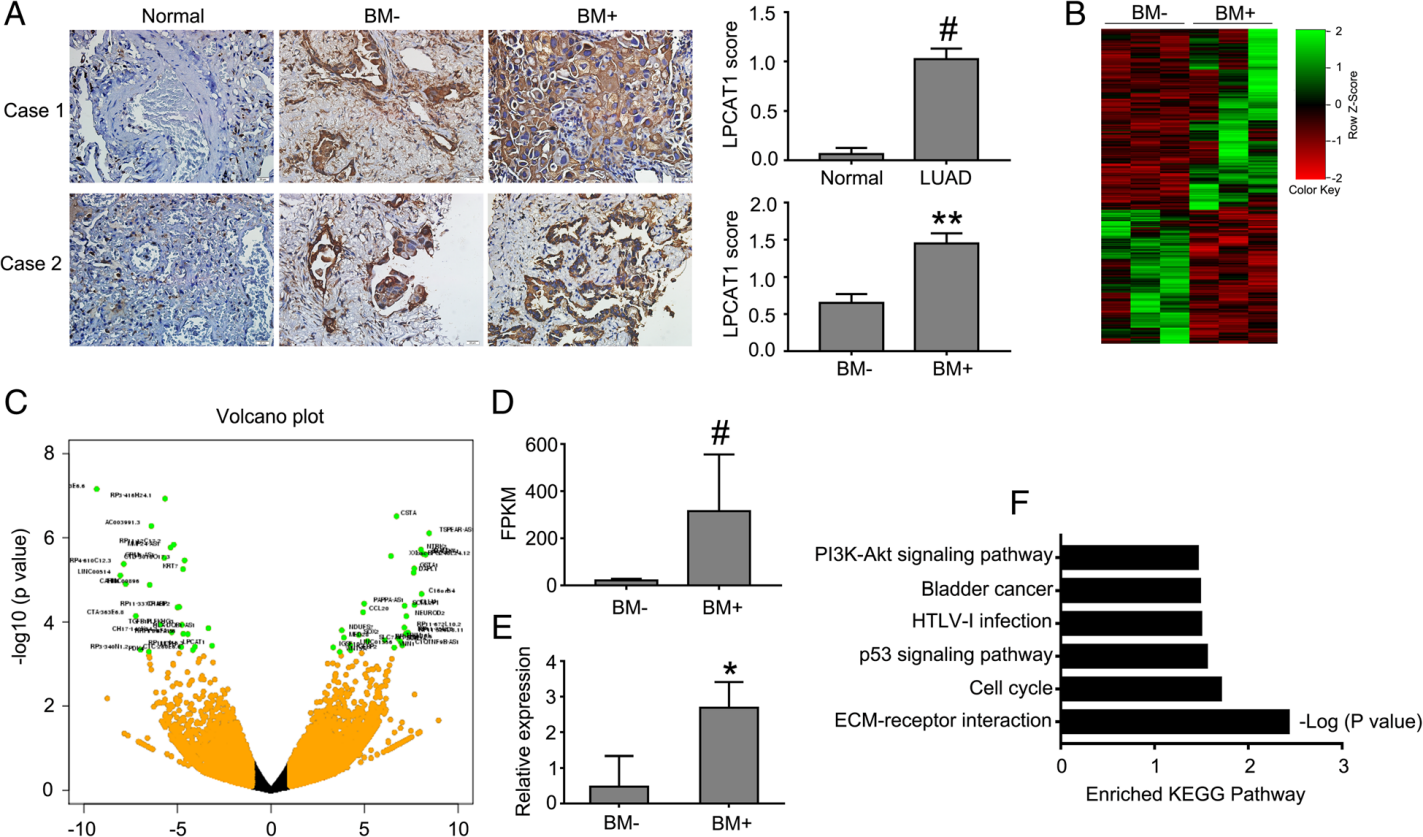
Lung tumors from patients with BM showed high LPCAT1 expression and PI3K/AKT/MYC gene signatures. **a** Representative IHC staining and quantitative analysis showing the expression of LPCAT1 in lung tumors from NSCLC patients with or without BM, and in normal lung tissues. **b** Heatmap of 566 differentially-expressed genes identified by RNA Sequencing with log2FoldChange more than 2 and *P* < 0.05 in primary lung tumor tissues from NSCLC patients with or without BM. **c** Volcano plot revealing the top 60 differentially-expressed genes. **d** The FPKM value of LPCAT1 expression from RNA-Seq data of BM- and BM+ lung tumor patients. **e** PCR analysis of LPCAT1 expression in the serum of BM- and BM+ NSCLC patients (*n* = 10). **f** Enriched KEGG pathway analysis of differentially expressed genes by DAVID. *P < 0.05, ^#^*P* < 0.001, as determined by the *t*-test

In order to confirm aforementioned findings and examine the association of LPCAT1 with brain metastases in NSCLC, we analyzed the LPCAT1 expression in primary tissues of NSCLC patients with or without BM and normal lung tissues by IHC staining (*n* = 5). Consistent with our findings in database analysis, LPCAT1 was found to be also considerably upregulated in primary tissues of NSCLC. Of note, the LPCAT1 expression was even higher in lung tumor tissues from NSCLC patients with BM in comparison to those from NSCLC patients without BM while the expression of LPCAT1 was virtually negative in normal lung tissues (Fig. 6a).

To further examine the above findings at the mRNA level, we analyzed our RNA-Seq datasets of lung cancer patients with brain metastases (BM+) and without brain metastases (BM-) to examine potential signaling pathways and gene signatures associated with brain metastases. Totally, 566 differentially-expressed genes between BM+ and BM- samples were identified by DESeq2 with log2Fold Change being more than 2 and *P* value of statistical test less than 0.05, as listed in Additional file 5: Table S4. A total of 326 genes were significantly down-regulated and 240 genes were up-regulated in BM+ group as shown in the heatmap (Fig. 6b). The top 60 differentially-expressed genes were highlighted in volcano plot (Fig. 6c). LPCAT1 was one of significantly up-regulated genes in BM+ group (Fig. 6d), suggesting LPCAT1 expression was associated with BM in NSCLC. Moreover, we performed RT-PCR to detect LPCAT1 expression in the serum of BM+ and BM- NSCLC patients (*n* = 10). The result indicated that LPCAT1 expression was up-regulated in BM+ group (Fig. 6e). Additionally, we performed KEGG pathway and DAVID GO analysis of those differentially-expressed genes and the results showed that this gene list was intimately associated with ‘ECM-receptor interaction’, ‘P53 pathway’ and ‘PI3K-Akt signaling pathway’ (Fig. 6f).

### High LPCAT1 expression was correlated with poor clinical outcome in NSCLC

**Fig. 7:**
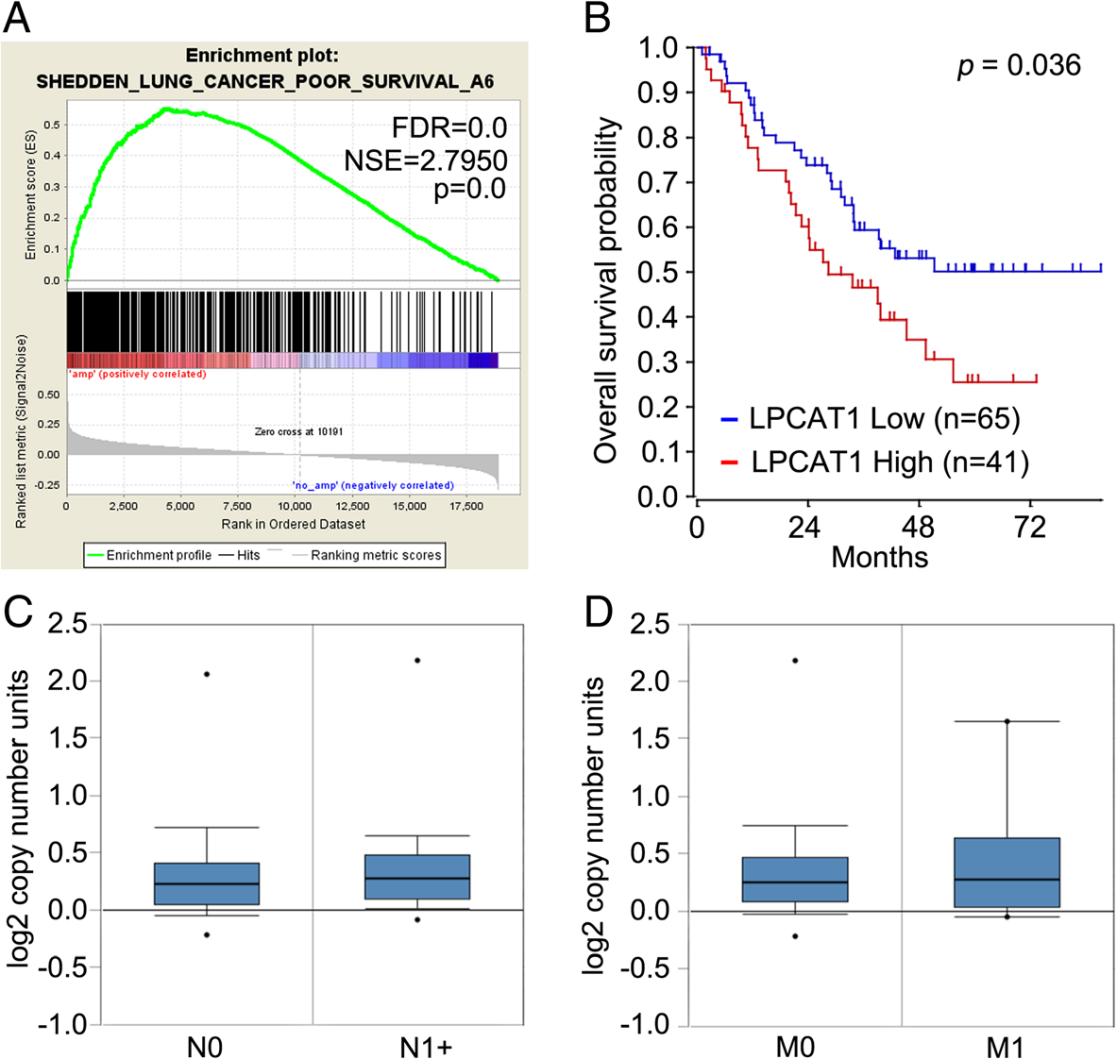
High LPCAT1 expression is correlated with poor clinical outcome in NSCLC. **a** GSEA analysis showed that overexpression of LPCAT1 was associated with poor outcomes of NSCLC. **b** Kaplan-Meier curves indicating the overall survival of LUAD patients with high or low LPCAT1 expression. **c**, **d** Association between the LPCAT1 expression and TNM stage of lung cancer

We further searched the R2 (https://hgserver1.amc.nl/cgi-bin/r2/main.cgi) and GSEA database, and found that overall survival of LUAD patients with LPCAT1 over-expression was significantly shorter than their counterparts with low LPCAT1 expression (Fig. 7a and b). However, no association was revealed between the LPCAT1 expression and TNM stage of lung cancer (Fig. 7c). Our survival results suggested that elevation of LPCAT1 might be involved in the carcinogenesis and brain metastasis of lung cancer.

## Discussion

Lung cancer represents one of the major life-threatening malignancies and BM is one of the principal causes of lung-cancer-related deaths. BMs are the leading cause of adult intracranial tumors, outnumbering primary brain tumors by ten-fold [30]. BMs are reportedly the primary or contributing cause of death in 50% of patients with BMs [31]. Among them, lung cancer accounts for 40–50% [30], and NSCLC is the most common metastatic tumor of the central nervous system [32]. Despite advances in cancer treatment, the median survival time of patients with BMs lasts only 9.3–19.1 months [33]. Identifying key target genes may help characterize the pathways involved in BM and pave the way to targeted therapies. In this study, we demonstrated that LPCAT1 was an important biomarker for NSCLC, especially BM. LPCAT1 was found to be highly expressed in NSCLC cells and lung tumors of NSCLC patients with BM. Over-expression of LPCAT1 was associated with poor prognosis of NSCLC. More importantly, we showed that LPCAT1 promoted growth and metastasis of NSCLC cells and was involved in the pathogenesis of NSCLC.

By using *in silicon* analysis of expression profiles of normal and lung adenocarcinoma tissues based on NCBI GEO and TCGA databases, we found that, compared to normal tissues, the expressions of 1220 differentially-expressed genes were all up-regulated in lung adenocarcinoma tissues. Particularly, we found that 15 cancer-related genes were much less studied. We specifically examined the expressions of the 15 candidate genes and found that, by analyzing genomic dataset of 522 LUAD patients from TCGA, LPCAT1 had the highest amplification rate. Moreover, patients with LPCAT1 over-expression had poor outcomes. Analysis of THPA revealed that LPCAT1 expression was relatively higher in lung cancer than in other 16 tumors, and the LPCAT1 expression in lung adenocarcinoma was significantly higher than in lung squamous cell carcinoma.

LPCAT1 is a cytosolic enzyme that catalyzes the conversion of LPC to PC in remodeling of the PC biosynthesis pathway. To date, LPCAT1 over-expression has been reported in hepatocellular carcinoma [34], colorectal adenocarcinoma [35], prostate cancer [36–38] and oral squamous cell carcinoma [12, 39], and has been identified as a contributor to cancer progression, metastasis, and recurrence. However, the role of LPCAT1 in NSCLC has not been well studied. Our study, for the first time, showed that LPCAT1 was up-regulated in NSCLC cells. By IHC analysis of samples from patients, we found that LPCAT1 expression was also up-regulated in NSCLC tissues and was substantially higher in lung tumor tissues from NSCLC patients with BM. Our results were consistent with the data from TCGA database, confirming that LPCAT1 was up-regulated in NSCLC tissues. We further studied the roles of LPCAT1 in the proliferation, migration, invasion of NSCLC cells and tumorigenesis and found that knockdown of LPCAT1 inhibited proliferation, migration, invasion of NSCLC cells and NSCLC tumorigenesis and induced G1 phase arrest. IHC analysis showed that LPCAT1 knockdown inhibited MMP-9 and CD34 expression in xenograft tumors. These findings suggested that LPCAT1 promoted NSCLC invasion and metastasis. Moreover, we found that LPCAT1 knockdown inhibited brain metastatic lesions in the mice model of brain metastasis.

PI3K pathway, which plays a key role in controlling cell proliferation, growth and survival, is activated in multiple cancers [40, 41]. Phosphorylated AKT may contribute to biological behaviors of cancer cells, such as viability [42], proliferation [43], invasion [44] and migration [45]. The PI3K/AKT signaling pathway can also induce the EMT, which has been generally considered to be an activator of cancer progression [46]. Moreover, one study reported that AKT was over-expressed in brain metastases compared with extracranial metastases, and unsupervised clustering analysis showed that PI3K/AKT pathway was tightly clustered, indicating that the pathway was activated [5]. In this study, we found that p-AKT expression was inhibited in NSCLC cells with LPCAT1 knockdown. When NSCLC cells were treated with exogenous IGF-1, an activator of PI3K/AKT pathway, the inhibition of p-AKT expression and cell proliferation was abolished in NSCLC cells with depletion of LPCAT1. These results indicated that LPCAT1 might exert pro-tumorigenic effects on NSCLC partially through PI3K/AKT pathway. GSEA analysis revealed that LPCAT1 was strongly enriched in the datasets of “MYC_UP.V1_UP” and “MYC_UP.V1_DN”, suggesting that MYC was activated when LPCAT1 was overexpressed. MYC, which is over-expressed in a variety of tumors, plays important roles in tumor proliferation, apoptosis and tumorigenesis [47]. Our results showed that MYC expression decreased in LPCAT1 knockdown group, and inhibition of MYC expression was reversed upon treatment of NSCLC cells with exogenous IGF-1. Moreover, co-IP assay showed that there was a definitive interaction between LPCAT1 and MYC. These results demonstrated that MYC might act as a downstream regulator of PI3K/AKT pathway, and LPCAT1 might promote NSCLC progression, at least in part, through the PI3K/AKT/MYC signaling pathway.

RNA-Seq analyses of various cancers have identified thousands of target genes that help guide clinical treatment. For instance, with Ewing sarcoma, some targets are found to mediate tumorigenesis, drug resistance and cell metastasis [48–50]. In this study, by using RNA-Seq analysis, we examined primary lung tumors from NSCLC patients with and without BM and identified differentially-expressed genes in two groups. Among these genes, LPCAT1 expression was significantly higher in BM+ group than in BM- group. PCR analysis of serum specimens from NSCLC patients with BM and without BM yielded similar results. Additionally, KEGG pathway and DAVID GO analysis of those differentially-expressed genes showed that this gene list was closely associated with ‘PI3K-Akt signaling pathway’. These results suggested that LPCAT1 can serve as a biomarker for the identification of the NSCLC patients with high risk for BM.

The current study focuses on the roles of LPCAT1 in EGFR-mutated NSCLC cells, but the effects of LPCAT1 in KRAS-mutated NSCLC cell lines (A549 and H460) have not been explored. In this study, we found LPCAT1 was highly expressed in A549 and H460 cells compared with the normal human bronchial epithelial cell line Beas-2B. Moreover, KRAS gene is an important downstream molecule in the EGFR signal transduction pathway [51], and KRAS is involved in proper stimulation of PI3K signaling cascade [52]. Previous studies also found that the expression of AKT and p-AKT was significantly higher in NSCLC tissue with KRAS mutation compared to those with wild-type KRAS [51, 53]. The PI3K/AKT pathway is involved in the proliferation and metastasis of cancers. Therefore, we speculated that LPCAT1 could promote KRAS-mutated NSCLC cells progress. However, the detailed effects and mechanism of LPCAT1 in KRAS-mutated NSCLC cells remains to be further elucidated in future study.

## Conclusions

In summary, our study demonstrated that LPCAT1 was up-regulated in NSCLC cells and tissues. LPCAT1 promoted NSCLC proliferation, metastasis and tumorigenesis both in vitro and in vivo and, LPCAT1 promoted NSCLC progression, in part, via the PI3K/AKT/MYC pathway. Therefore, LPCAT1 might serve as a target for the NSCLC treatment.

## Additional files

Additional file 1: Table S1. Differentially-expressed genes from LUAD patients and normal patients in GSE32863, GSE7670 and TCGA-LUAD datasets, respectively. (XLSX 2088 kb)

Additional file 2: Table S2. Differentially-expressed genes in lung adenocarcinoma tissues against normal tissues among TCGA, GSE32863 and GSE7670 datasets. (XLSX 25 kb)

Additional file 3: Table S3. KEGG and GO analysis related to biological progresses, molecular functions and biological cellular components. (XLSX 127 kb)

Additional file 4: Figure S1. Representative IHC staining analysis of LPCAT1 in xenograft tumors (**a**) and brain metastasis tumors (**b**) (200×). (DOCX 286 kb)

Additional file 5: Table S4. Differentially-expressed genes between BM+ and BM- samples identified by DESeq2 (log2Fold Change>2). (XLSX 87 kb)

## Abbreviations

ATCC: American Type Culture Collection
BM: Brain metastasis
CCK-8: Cell counting kit-8
DAVID: Database for Annotation, Visualization and Integrated Discovery
DEGs: Differentially expressed genes
GEO: Gene Express Omnibus
LPC: Lysophosphatidylcholine
LPCAT1: Lysophosphatidylcholine acyltransferase 1
LUAD: Lung adenocarcinoma
NSCLC: Non-small cell lung cancer
PC: Phosphatidylcholine
shLPCAT1: Small hairpin RNA (shRNA) specific for LPCAT1
shNC: The negative control shRNA-luciferase
TCGA: The Cancer Genome Atlas
THPA: The Human Protein Atlas
