# Supplementary material for: LPCAT1 promotes brain metastasis of lung adenocarcinoma by up-regulating PI3K/AKT/MYC pathway
Source: J Exp Clin Cancer Res. 2019 Feb 21;38:95. doi: 10.1186/s13046-019-1092-4 (PMC6385475; doi:10.1186/s13046-019-1092-4)
Supplement: Supplementary file 4 — Representative IHC staining analysis of LPCAT1 in xenograft tumors (a) and brain metastasis tumors (b) (200×). (DOCX 286 kb) [file 13046_2019_1092_MOESM4_ESM.docx]

**Methods**

**Immunohistochemical quantitative analysis**

Independent and non-repeating pictures were taken from each tissue slice for the quantification. Before scoring the images, IHC Profiler (https://sourceforge.net/projects/ihcprofiler/) plugins was installed on the ImageJ Software (https://imagej.nih.gov/ij/). As a general procedure, images were opened in ImageJ, followed by deconvolution used the newly optimized color deconvolution plugin. With the selection of the ‘H DAB’ vector on the color deconvolution popup window, IHC profiler automatically ploteds a histogram profile of the DAB image and the corresponding scoring log was displayed on the screen as followed. The flow chart representing the functioning of theworking algorithm designed for the macro is shown as follows. Images were assigned a score as negative (0), low positive (1+), positive (2+), and high positive (3+) by using ImageJ Software. QuantitativeQualitative data for each image was used for subsequent statistical analysis.


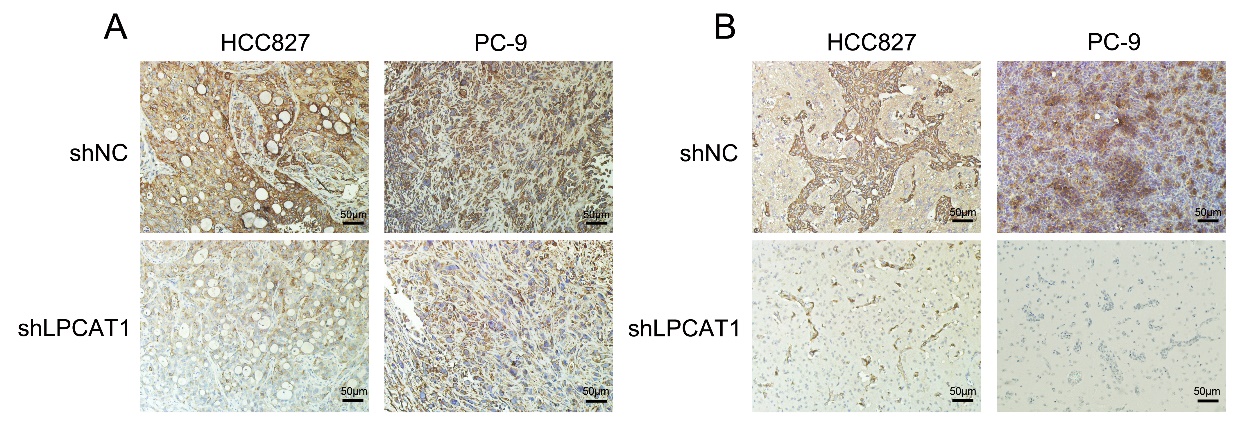


**Figure S1.** Representative IHC staining analysis of LPCAT1 in xenograft tumors (**a**) and brain metastasis tumors (**b**) (200×).
